# The conditional benefit of being alive and disease free at 2 years after transplant: a long term survival evaluation among 456 patients affected by acute myeloid leukemia

**DOI:** 10.3389/fimmu.2026.1818603

**Published:** 2026-05-19

**Authors:** Alessandro Bruno, Francesca Lorentino, Matteo G. Carrabba, Sara Mastaglio, Raffaella Greco, Sarah Marktel, Simona Piemontese, Magda Marcatti, Luca Vago, Francesca Farina, Daniela Clerici, Elisa Diral, Lorenzo Lazzari, Edoardo Campodonico, Daniele Sannipoli, Andrea Assanelli, Consuelo Corti, Massimo Bernardi, Jacopo Peccatori, Fabio Ciceri, Maria Teresa Lupo-Stanghellini

**Affiliations:** 1 Hematology and Bone Marrow Transplantation Unit, IRCCS San Raffaele Scientific Institute, Milan, Italy; 2 Vita-Salute San Raffaele University, Milan, Italy

**Keywords:** allogeneic stem cell transplantation (allo-SCT), AML - acute myeloid leukemia, disease status at transplantation, long term survivorship, overall survival

## Abstract

Risk factors for relapse and non-relapse mortality (NRM) in the first years after allogeneic hematopoietic stem cells transplant (allo-HSCT) for acute myeloid leukemia (AML) are well known, but their correlation with long-term survivorship is unclear. This study aims to evaluate factors that impact on long-term survival after allo-HSCT in AML. We retrospectively analyzed data of 456 consecutive patients who received a first allo-HSCT in our center, regardless of conditioning intensity, GvHD prophylaxis, donor matching and HSC source. At transplant, two hundred and four patients (44%) were in CR1, 62 patients (14%) in CR>1, 27 (6%) in CR MRD+ and 163 patients (36%) in active disease (AD). Two-hundred and thirty-nine patients (52%) were alive 2 years after allo-HSCT. Median follow-up was 6.1 years. The 5 years OS was 63% for CR1, 38% for CR>1 and 25% for AD. However, in patients alive and disease free 2 years after allo-HSCT the probability of being alive for 3 more years was 86% for CR1, 72% for CR>1 and 79% for AD, with no statistically significant differences between CR>1 and AD. Other variables associated with better outcomes included younger age, more recent transplantation, and having a female donor for a male recipient. Relapse remained the main cause of death, even among long-term survivors. In conclusion, the negative impact of advanced disease at the time of allo-HSCT appears to progressively reduce after transplantation, highlighting the excellent long-term outcomes of patients who remain disease-free at 2 years after allo-HSCT, regardless of pre-transplantation disease status.

## Introduction

Acute myeloid leukemia (AML) is the most frequent indication for allogeneic hematopoietic stem cells transplantation (allo-HSCT), accounting for 39% of allo-HSCT performed in Europe in 2022 (1). European LeukemiaNet guidelines recommend allo-HSCT in fit patients with intermediate and adverse risk AML or with favorable risk AML with positive measurable residual disease (MRD) in first complete remission (CR1) or in any risk category if in CR2 (2). Allo-HSCT may be useful as a rescue treatment in patients with primary induction failure (PIF) or those with active disease (AD) (3), with reports showing a clear benefit even in older patients (> 60 years) (4). Nevertheless, disease status at transplantation is still one the main factors predicting survival after allo-HSCT. Patients who undergo allo-HSCT in MRD negative CR1 have the longest overall survival (OS) and the lowest relapse rates, with outcomes progressively worsening in MRD positive CR1, CR2 or higher, and AD (5–7). Several factors related to patient, donor and disease are considered when evaluating allo-HSCT as a treatment option. For example, patient’s chronological age has been replaced by comprehensive geriatric assessments (IADL (8), HCT-CI (9)), while strategies for allo-HSCT from alternative donors are rapidly evolving with encouraging results (10–13). Outcomes of allo-HSCT are steadily improving, mainly because of the reduction of non-relapse mortality (NRM) (14, 15), with a significant proportion of patients becoming long-term survivors.

The definition of long-term survivorship after allo-HSCT is not unanimous. In a report from Pond et al., only 62% of patients survived the first year after allo-HSCT, but 98.5% of those that were alive after 6 years survived at least another year, with 31% of the deaths in long-term survivors considered unrelated to transplantation or relapse. The difference in life expectancy between the two groups decreased the longer the patients survived (16). In another report, patients who survived free of the original disease for at least 5 years after allo-HSCT had a high probability of surviving for an additional 15 years; however, life expectancy was never completely restored. Main causes of late excess deaths were second malignancies and recurrent disease, followed by infections and graft-versus-host disease (GvHD) (17). In a historical cohort from CIBMTR, the probability of being alive for 5 or more years after achieving 2 disease-free years following allo-HSCT was 89%. The main causes of death in long-term survivors were disease relapse, GvHD, and second cancers (18).

Another large worldwide analysis of 4017 patients with AML who underwent allo-HSCT between 1980 and 2003 and were alive at 2 years after allo-HSCT showed 84% OS, 10% cumulative incidence of relapse and 9% NRM at 10 years after allo-HSCT. Disease relapse was confirmed as the most common cause of death. In multivariate analysis, risk factors for late death were older age, chronic GvHD occurring before 2 years, and advanced disease at transplantation (19).

In this study we analyzed prognostic factors for long-term survival after allo-HSCT in AML patients, with a focus on disease status at transplantation. We evaluated outcomes of patients who were alive and disease free at 2 years after allo-HSCT given the significant reduction in relapse risk (20) and the increasing likelihood of becoming long-term survivors beyond this time point. Focusing on this selected population of survivors we recognize the peculiarity of this cohort, highlighting the importance of a tailored follow-up in the early years post-transplant as well as in the long term follow-up.

## Methods

We retrospectively analyzed 456 consecutive patients with AML who underwent first allo-HSCT between February 2004 and December 2019 in our center, regardless of disease status at allo-HSCT, conditioning intensity, GvHD prophylaxis, donor matching and stem cells source. The primary aim of this study was to determine factors for long-term survivorship in patients with AML alive after 2 years from allo-HSCT. The probabilities of progression-free survival (PFS), defined as the length of time from allo-HSCT to disease progression, and OS, defined as the length of time from allo-HSCT to death, were estimated using the Kaplan-Meier method. The risk factors for transplantation outcomes were selected after revisions of the literature. We consider risk factors classically associated with transplant outcome: age and disease status at transplantation (assuming that older patients age is associated to worse outcome as well as persistence of disease), year of a transplant (assuming that transplant performed in a less recent period would be associated with a worst outcome), donor type (match related vs match unrelated vs mismatch related or unrelated) and donor/recipient sex mismatch, stem cell source (peripheral blood vs bone marrow vs cord blood), conditioning intensity, GvHD prophylaxis (namely use of ATG vs PT-Cy). The following variables were considered in multivariate COX model: median age (53 y), median year of allo-HSCT (2014), use of ATG, female donor to male recipient, stem cell source, disease status at allo-HSCT (CR1 vs AD and CR>1 vs AD) and secondary AML. Other variables, such as donor type, were excluded due to not meeting the proportional hazards assumption. Potential risk factors were analyzed in the overall study population and using a 2-year landmark analysis from allo-HSCT in patients who were alive and disease-free at this time point. For statistical analysis, CR MRD+ patients were considered to have active disease given that most had positive cytogenetic disease markers, regardless of CR number. Patients who underwent allo-HSCT with a defined program of prophylactic maintenance treatment were excluded from the cohort to avoid a potential bias. Hazard ratios for which the 95% CI included 1.0 were considered not statistically significant (p = 0.05). Statistical analysis was performed using R version 4.3.

## Results

**Figure 1:**
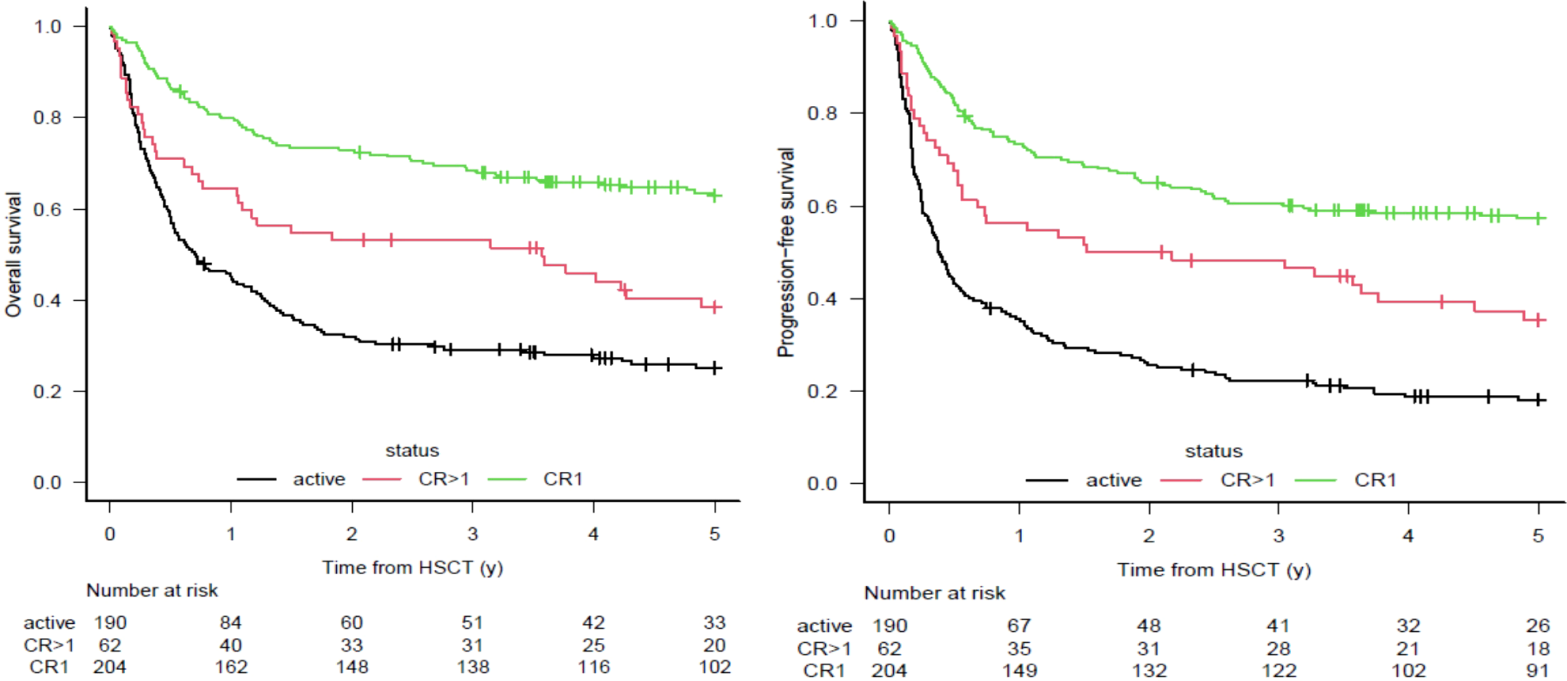
Main outcomes. Kaplan-Meier curves for Overall Survival (OS) and Progression Free Survival.(PFS) in the overall study population.

**Figure 2:**
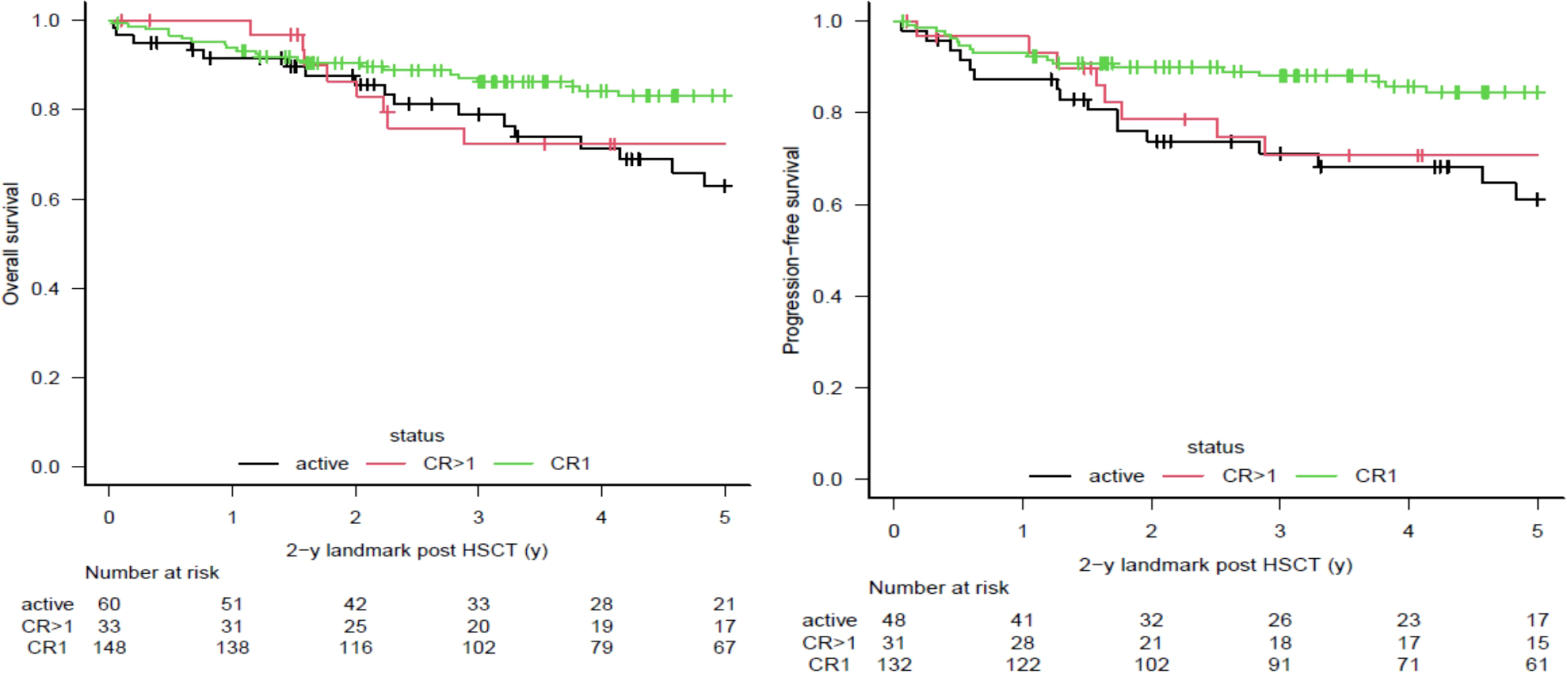
Main outcomes. 2 years landmark analysis - Kaplan-Meier curves for Overall Survival (OS) and Progression Free Survival.(PFS).

The characteristics of the analyzed patients are summarized in **Table 1**. Overall median follow-up was approximately 2.7 years, while median follow-up for patients surviving at least 2 years after allo-HSCT was approximately 6.1 years. One hundred ninety patients (42%) were alive at last follow-up. The overall causes of death were progressive disease in 125 (27%), treatment-related causes in 132 (29%), and unknown causes in 9 cases (2%). Two-hundred thirty-nine patients (52%) were alive and disease free at 2 years after allo-HSCT. Among patients alive at this time point, 51 (21%) died during subsequent follow-up: 31 from progressive disease (13%), 18 from treatment-related complications (8%), and 2 from unknown causes (1%). Fifteen patients were lost at follow-up (6%). Five-years OS for the entire cohort was 0.63 (95% CI 0.59-0.69) for patients in CR1, 0.38 (95% CI 0.26-0.51) for patients in CR>1, and 0.25 (95% CI 0.19-0.32) for patients in AD. Five-years PFS was 0.57 (95% CI 0.50-0.64) for patients in CR1, 0.35 (95% CI 0.23-0.47) in patients in CR>1, and 0.18 (95% CI 0.13-0.24) for patients in AD (**Figure 1**). For the 239 patients alive and disease free 2-year after transplant, a landmark analysis was performed providing the OS and PFS at the subsequent 3 years (overall 5-year after transplantation). At 2-year landmark analysis, the subsequent 3-years OS was 0.86 (95% CI 0.79-0.91) for patients in CR1, 0.72 (95% CI 0.52-0.85) for patients in CR>1 and 0.79 (95% CI 0.65-0.88) for patients in AD. Accordingly, the subsequent 3-years PFS was 0.88 (95% CI 0.81-0.93) for patients in CR1, 0.71 (95% CI 0.50-0.84) in patients in CR>1 and 0.711 (95% CI 0.55-0.82) for patients in AD (**Figure 2**). In the overall cohort, lack of use of ATG, allo-HSCT performed after 2013, female donor to male recipient, age at allo-HSCT <53 y and being in CR1 all resulted significantly linked to better OS, PFS, NRM and relapse risk. In landmark analysis, less significant risk factor were found: age below median and being in CR1 were the factors most consistently associated with improved OS, PFS and NRM, and reduced relapse risk. Being in CR>1 did not improve OS, PFS, and NRM, and did not reduce relapse risk in comparison to AD (**Table 2**).

**Table 1:** Patients' characteristic.

*Variable*	*No. of patients (total = 456)*
*Male sex* *Median Age*	263 (58%) 53 y (range 18-78y)
*sAML*	64 (14%)
*Disease status* *CR1* *CR>1* *CR MRD+* *AD*	204 (44%) 62 (14%) 27 (6%) 163 (36%)
*Donor* *Sibling* *MUD* *MMRD* *CBU*	96 (21%) 120 (26%) 221 (49%) 19 (4%)
*Source* *BM* *PBSC*	20 (4%) 417 (92%)
*Conditioning* *Treosulfan-based* *Busulfan-based* *Other* *MAC*	391 (86%) 41 (9%) 24 (5%) 233 (51%)
*GvHD prophylaxis* *PT-Cy* *ATG + other* *Sirolimus*	182 (40%) 274 (60%) 296 (65%)

CR, complete remission; AD, active disease; MUD, matched unrelated donor;MMRD, mismatched related donor; CBU, cord blood unit; BM, bone marrow;PBSC, peripheral blood stem cells; MAC, myeloablative conditioning;PT-Cy, post-transplant cyclophosphamide; ATG, anti-thymocyte globulin.

**Table 2:** COX analysis at 2 years landmark.

*OS*				
	Hazard ratio	Lower 95%CI	Upper 95%CI	p.value
*Disease status [CR>1 vs AD] *	1.062	0.511	2.205	0.872
*Disease status [CR1 vs AD] *	0.434	0.234	0.806	0.008
*Age (≥53)*	1.797	1.033	3.125	0.037

*PFS*				
	Hazard ratio	Lower 95%CI	Upper 95%CI	p.value
*Disease status [CR>1 vs AD]*	0.847	0.387	1.852	0.678
*Disease status [CR1 vs AD]*	0.332	0.172	0.641	0.001
*Age (≥53)*	2.099	1.155	3.817	0.015

*NRM*				
	Hazard ratio	Lower 95%CI	Upper 95%CI	p.value
*Disease status [CR>1 vs AD]*	1.126	0.342	3.702	0.844
*Disease status[CR1 vs AD]*	0.302	0.101	0.904	0.032
*Age (≥53)*	4.044	1.425	11.480	0.008

*Relapse*				
	Hazard ratio	Lower 95%CI	Upper 95%CI	p.value
*No risk factors*				

## Discussion

Nowadays, allo-HSCT represents an option of cure for several patients affected by AML. Major achievements have been reached thanks to the advent of less toxic conditioning regimen as well as more efficacious strategies of GvHD prophylaxis and, most of all, optimization of donor selection. Improving results are being achieved thanks to a reduction in NRM through the improvement of supportive therapies, as well as a decrease in disease relapse through a tailored program of post-transplantation prophylactic strategies. Of note, reports often focus on early outcome after transplantation, mostly before the 2 years landmark, addressing typical events of this time window. This results into poor information on long-term survival. Recently the MDACC group (21) focused the attention on long-term survival outcome for patients alive and disease free 2 years after a haploidentical allo-HSCT highlighting three major goal: the outcome for patients alive and in remission 2 years after allo-HSCT is excellent with a very low rate of relapse and NRM, age (>/< 55 years) was the only predicting factor for both OS and PFS, and disease status was not impacting on both OS and PFS for patients alive and in remission 2 years after allo-HSCT. This last result points out that the “disease effect” mainly affects the early phase after allo-HSCT. Few other publications have focused on conditional survival to evaluate the long-term survival expectancy in the past 25 years. Socié et al. published in 1999 the results (18) of long-term survival for 6500 patients alive 2 years after transplantation. The authors for the first time stated that these patients could be considered cured from their primary diseases (expected 5 years OS: 89%), even though the mortality was still higher than that in the normal population. Notably, median age at transplantation for this cohort of patients was 25 years (range <1-69y), 70% of patients was in early disease status at transplantation and 87% received a transplantation from an HLA identical sibling. In 2010, Martin and colleagues reported the long-term survival results (17) of a cohort of more than 2500 patients alive and in remission 5 years after allo-HSCT at FHCRC: median age at transplantation was 32 years and the majority of patients (63%) received a transplantation from an HLA identical sibling. The authors underscored how patients who have survived for at least 5 years after allo-HSCT without recurrence of the original disease had a high probability of surviving for an additional 15 years (20 years OS: 80%), even though life expectancy was not fully restored (with an excess of mortality in comparison with the general population) mainly due to treatment-related late complications. Similarly, Wingard and coauthors reported on 2011 the analysis (19) based on more than 10.000 patients registered in the CIBMTR registry. This cohort was also mainly representative of young patients (median age <35 years) transplanted in early disease status and with a clear prevalence of an HLA identical sibling. Moreover, the prospect for long-term survival was excellent for 2-year survivors of allo-HSCT (10 years OS: 85%), even though, again, life expectancy remained lower than expected. In summary, as confirmed by the recent MDACC report, this three large studies support the idea of a breakthrough in the probability of post-transplantation survival for patients alive and in remission 2 years after transplantation.

The general analysis of our dataset shows slightly worse OS and PFS compared to these three large studies. The higher proportion of allo-HSCT performed in advanced disease status, as well as the older age at transplantation may have potentially contributed to a less brilliant overall outcome (17–19). Indeed, when stratified by disease status at transplantation (**Figure 1**), our OS and PFS were comparable to those reported previously. Notably, in the landmark analysis stratified by disease status at transplantation, OS and PFS were similar across disease categories: patients transplanted in CR1 had only slightly better outcomes compared with those in CR>1 and AD, between whom no differences were observed. These results confirm the observation of the MDACC group: disease status exerts its main effect in the early months after transplantation. The expectancy of long term survival remain consistent for patients alive and in remission 2 years after allo-HSCT, even for those with dismal prognosis due to unfavorable disease features at transplantation: for patients in CR1 the 5-years overall survival exceed the 80% if alive and disease free at 2-year after transplant, while the 5-years survival exceed the 70% among patients in CR>1 or in AD. We can postulate that the establishment of an effective graft-versus-leukemia effect after allo-HSCT is fundamental and reaching a 2-year survival confers high probability of further long-term survival, even in patients with advanced disease at transplantation. For these reasons, we believe that all available tools, such as maintenance or preemptive treatments, need to be considered in intermediate and advanced disease patients, while we hope that ongoing drug trials will prove effective and support their use.

We also noted that, in the general cohort, NRM was comparable between CR>1 and AD. Similar results are already reported in literature (22) and possibly related to longer disease history and a greater burden of previous toxic treatments. The prognostic importance of age at transplantation is confirmed in our analysis, in which median age (53 years) was used as reference. In particular, younger patients have a benefit in both OS, PFS and NRM in the overall cohort. Age retains a significant impact in all the landmark analysis for NRM advocating for less toxic conditioning regimen. Moreover, younger patients should be considered for intensified treatment to pursue the goal of transplantation in the better disease status and to target the goal of complete remission at 2 years after allo-HSCT.

*Vice versa*, proceeding to allo-HSCT is useful to obtain long-term disease control in a significant part of patients who are refractory or relapse after conventional induction treatment and in which remission is therefore not obtainable before transplantation. This is especially true for those young and fit patients who are supposed to have a lower NRM (23–25). Other statistically significant variables associated with improved OS and PFS were lack of ATG use and allo-HSCT performed in or after 2014, but were not confirmed in landmark analysis. Considering the progressive reduction of ATG use in our center in favor of PT-Cy, these variables seem to be related and their impact is mostly limited to early mortality both for NRM or early relapse. In general, they probably just reflect the continuous improvement in supportive care. In the general cohort, having a female donor for a male recipient was associated with better PFS and less relapse risk, while this was not confirmed in the landmark analysis. This observation was already reported in the literature (26) and possibly related to stronger GvL with female donors in the first months after allo-HSCT. As already known in previous reports (27), the most common cause of death after 2 years from allo-HSCT is still disease relapse: of the 239 patients who were alive at 2 years, 31 (13%) eventually relapsed and most of them (84%) died because of the disease itself or because of further treatment complications. AML relapse is still a dreadful event with poor prognosis even in the era of venetoclax and targeted therapies (27–29). In our analysis, severe prognosis is confirmed for patients relapsing after 2 years from allo-HSCT, in a similar fashion to patients relapsing before 2 years. Several studies are ongoing to clarify the biology of AML relapse and in particular of later ones. Within the GITMO (Gruppo Italiano Trapianto di Midollo Osseo) we strongly support a dedicated research program to better clarify mechanism of disease relapse after allo-HSCT: by integrating biological information and clinical data using the means of machine learning algorithms, we aim to identify associations between transplantation variables, relapse patterns, and response to salvage therapies (NCT05124288).

In conclusion, traditional transplant-related variables that strongly influence early outcomes, such as more advanced disease status, seem to have a reduced prognostic impact after the first 2 years post transplantation, while the patient’s clinical condition is likely to play a more prominent role in determining the risk of mortality and relapse thereafter. Patients who remain disease-free 2 years after allo-HSCT constitute a distinct, selected subgroup characterized by a “survivor profile”, and as such are not fully representative of the overall population initially undergoing transplantation. These observations need to be validated and confirmed in multicenter larger studies. However, they have great significance in patients’ counseling, where they allow a clearer understanding of the rationale for maintaining a regular long-term follow-up, even in cases not classically considered at high risk. Moreover, they highlight the need to implement maintenance strategies, which are increasingly feasible with the advent of novel therapies, in order to achieve the critical milestone of 2 years alive and disease-free from transplantation.

## Data Availability

The raw data supporting the conclusions of this article will be made available by the authors, without undue reservation.
